# Ship Detection in Gaofen-3 SAR Images Based on Sea Clutter Distribution Analysis and Deep Convolutional Neural Network

**DOI:** 10.3390/s18020334

**Published:** 2018-01-24

**Authors:** Quanzhi An, Zongxu Pan, Hongjian You

**Affiliations:** 1 School of Electronic, Electrical and Communication Engineering, University of Chinese Academy of Sciences, Huairou District, Beijing 101408, China; anquanzhi16@mails.ucas.ac.cn (Q.A.); hjyou@mail.ie.ac.cn (H.Y.); 2 Institute of Electronics, Chinese Academy of Sciences, Beijing 100190, China; 3 Key Laboratory of Technology in Geo-spatial Information Processing and Application System, Beijing 100190, China

**Keywords:** ship detection, Gaofen-3, fully convolutional network, truncated statistic, iterative censoring scheme, SAR applications, deep convolutional neural network

## Abstract

Target detection is one of the important applications in the field of remote sensing. The Gaofen-3 (GF-3) Synthetic Aperture Radar (SAR) satellite launched by China is a powerful tool for maritime monitoring. This work aims at detecting ships in GF-3 SAR images using a new land masking strategy, the appropriate model for sea clutter and a neural network as the discrimination scheme. Firstly, the fully convolutional network (FCN) is applied to separate the sea from the land. Then, by analyzing the sea clutter distribution in GF-3 SAR images, we choose the probability distribution model of Constant False Alarm Rate (CFAR) detector from K-distribution, Gamma distribution and Rayleigh distribution based on a tradeoff between the sea clutter modeling accuracy and the computational complexity. Furthermore, in order to better implement CFAR detection, we also use truncated statistic (TS) as a preprocessing scheme and iterative censoring scheme (ICS) for boosting the performance of detector. Finally, we employ a neural network to re-examine the results as the discrimination stage. Experiment results on three GF-3 SAR images verify the effectiveness and efficiency of this approach.

## 1. Introduction

As one of the most effective means of Earth observation, synthetic aperture radar (SAR) has gained widespread attention recently. In particular, ship detection is an important one when it comes to marine surveillance. A mature ship detection system usually consists of three necessary steps: land masking, prescreening, and discrimination [1]. The land masking stage distinguishes sea from land and defines a scope to be detected for subsequent steps. In the process of prescreening, certain approaches are used to search for potential target pixels throughout the whole image, among which CFAR is one of the most prevalent by virtue of its convenience and reliability, whose core is based on the sea clutter modeling to find bright pixels. It is worth noting that CFAR detector will inevitably introduce some false alarms, so the stage named discrimination is employed for false alarm elimination as a necessary supplement to the CFAR.

Although some work has been devoted into the ship detection in SAR image, there are also some key questions related to GF-3 SAR images. Before the process of target detection, sea-land segmentation is a necessary step because detected targets on the land are obvious mistakes. Except for this reason, land masking also has a significant influence on sea clutter modeling and the accuracy of ship detection, so an approach to the sea-land segmentation is necessary. In many previous studies, a pre-computed sea-land map is used to register the SAR image, which cannot be used in practice on account of its unsatisfactory accuracy [1]. In order to strengthen the performance of the method, a buffer zone around the coastline is used to prevent ships from being masked out [2,3]. This method is feasible but the buffer zone can’t be fundamentally eliminated. Furthermore, the coastline of the earth is constantly changing, so the use of fixed coastline data will inevitably result in some deviations. As another way of land masking, Ferrara and Torre [4] design an automatic system to detect coastlines, in which an edge operator is utilized to identify the position of the boundary. They claim to have obtained the right result but it should also be noted that this system is knowledge-based, requiring lots of prior knowledge which may be a potential bottleneck. Another kind of segmentation approach is based on mature mathematical theory. Markovian random field is applied to detect the coastlines in [5,6], comparing the pixels to their surrounding and generating a rough coastline. In [7], an algorithm for land masking is designed for ship detection in SAR images based on a series of image processing steps using mathematical transformation. The accuracy of the sea-land segmentation can be improved by evaluating the radiometric intensity gradient in zone closed to the sea in SAR image [8]. Experimental results from real SAR images demonstrate the usefulness of methods of wavelet transform [9,10] and threshold techniques [11,12]. Compared with earlier methods, such approaches do not require geography prior knowledge, but are necessary for complex mathematical theory. In [13], a kernel principal component analysis (KPCA) is utilized to obtain discriminative texture features for efficient sea ice segmentation, in which model the noise is considered to obey the Gaussian distribution. This kind of texture classification requires setting the size of the data block first, and selecting the appropriate kernel function according to the various data. Different from the hand-craft features, neural networks are utilized in our essay to achieve automatic extraction of features. In recent years, the development of computer vision technology also creates new ways of addressing image segmentation issue, especially deep learning methods. In comparison to traditional approaches [5,6,14], the use of fully convolutional network is a new attempt for image semantic segmentation and has achieved encouraging results [15]. In this paper, we propose a method of using the fully convolutional network to segment the land and sea in GF-3 SAR images, which avoids the artificial selection of the image features and is not affected by geographical changes. The reliable segmentation consequence lays the foundation for subsequent work.

After the land masking stage, CFAR-based methods are widely utilized to detect ships in the sea. The advantage of CFAR-based methods is their reliability and high efficiency. In [16], a maritime ship detection algorithm based on the Bayesian framework and effective enhancement methods is proposed for improving the detection performance. This algorithm realizes the accurate classification of pixels at the expense of more running time owing to the iterative calculation for all pixels. Different from the algorithm proposed in [16], our method replaces iterated conditional model with CFAR-based detector, which is more advantageous in computing efficiency. In the process of CFAR detection, an ideal distribution is chosen and subsequently an adaptive threshold is computed associated with a given constant false alarm rate. What significantly affects the detected result is which probability distribution models the actual situation better. A classic model is the Gaussian distribution, as the central limit theorem guarantees the rationality of this distribution [17]. It is often used in local sea clutter description to improve computational efficiency because of its simple form. Nevertheless, the Gaussian distribution cannot generate a precise model when the resolution is high. Later work finds that the choice of appropriate distribution is related to the characteristics of sea clutter. In [18], it is found that in SAR images with different sight angles, sea clutter has different characteristics, while the Weibull distribution and K-distribution show excellent fitting performance to sea clutter when the radar sight angles are variant. In [19], the gamma distribution is exploited to describe the texture of sea clutter in heterogeneous background, which demonstrates the usefulness of the CFAR detection with a simplified detection system. In [20], a maximum likelihood generalized gamma (MLGG) CFAR detector is used to estimate the parameters of the sea clutter. Higher robustness and reliability are guaranteed by introducing K-distribution for the sea clutter in [21], and the performance analysis on some remote sensing images is carried out to confirm the merits of this approach in reducing false alarm rate under variant circumstance. The Rayleigh distribution is usually used as a simplified distribution for CFAR detection, and the detection systems with Rayleigh distribution shows robustness when dealing with real data [22]. In fact, the choice of probability distribution is also different on account of various SAR satellites. Wackerman uses CFAR detector for both Gaussian and exponentially distributed sea clutter to deal with the Radarsat-1 C-band imagery, and the result reveals that the Gamma distribution may be a better choice [2]. For SIR-C L-band SAR imagery, the gamma distribution is a good choice, and chi-squared distribution is employed for the parameter estimation [4,23]. Gagnon uses a Rayleigh CFAR detector to cope with the imagery of stripmap SAR and achieves a well performance when the images have a high resolution [24]. It can be concluded that the choice of probability distribution is closely relevant to the characters of sea clutter in various SAR images. Hence the sea clutter distribution analysis is meaningful for detecting the targets in the imagery of new SAR satellites. In this paper, we analyze the distribution of sea clutter in GF-3 SAR images and give a comparison of several classical distributions. This work provides a reference for the applications of GF-3 SAR images, in especial maritime surveillance.

When the distribution of sea clutter is selected, the pixels labeled as sea clutter are used to estimate the distributed parameters. Ensuring that the sea clutter pixels used to estimate the parameters do not blend with target pixels is a guarantee of the parameters’ accuracy. Considering that the target pixels are generally brighter than the sea clutter, a truncated statistic (TS)-based detector is applied in [25,26], in which a fixed truncated ratio is employed to separate the potential ship pixels which have high brightness from the background pixels. The CFAR detectors based on TS prove to be robust in multi-target situation and are widely applicable [26]. In addition to removing the interference of the target pixels with a fixed ratio, using an iterative censoring scheme which updates the detection results iteratively also improves the performance in [27,28,29,30]. The research illustrates that the censoring-based methods are utilized for robust detection in complicated circumstances. To summarize, the choice of probability distribution influences the accuracy and speed of ship detection, so these two aspects should be taken into consideration to make the detector more practical. Additionally, the TS and iterative censoring scheme (ICS)-based detectors have a stronger robustness, boosting the accuracy of the parameter estimation and improving the performance of detection, consequently.

The discrimination stage is designed to remove false alarms so that most pixels that have been misdiagnosed as targets are removed after this stage. Discrimination algorithms usually operate on image chips which include the real ship targets and the false targets similar to the real ones [1]. In early years, the discrimination algorithm merely relies on some simple measurements, for example, using area, length, width and orientation information of the potential targets, etc. In general, it is common to reject such potential target either too small or too large. In [31], the detection results with a length more than 250 m are rejected when the CFAR detectors search for small fishing ships. These methods are more than simple to use with a fast processing speed, but the accuracy often cannot match the requirements. So the Ocean Monitoring Workstation (OMW) of Canada uses human supervision [32], the standard of which is also rejecting the objects which are too little, too big, too close to other potential targets or reefs [32,33]. Although the accuracy is guaranteed, this detection system requires an army of manpower and time. Thus, the research turns to eliminate false alarms in the process of detection. In [4], a link between prescreening and discrimination is established and discriminates the false alarms in the process of detection. Ferrara and Torre use two different false alarm rates in the prescreening stage so that the two results can complement each other and a higher accuracy rate is achieved. In addition to the analysis of the results of the CFAR algorithms, some work related to the wakes detection has also been carried out. Although the appearance of wakes is an uncertain event, an approach based on wakes detection is applied to confirm the appearance of some small ships [34]. Research also demonstrates that wakes without ships are infrequent in SAR imagery [34]. However, except the methods of detecting wakes, there is not much effort on the stage of discrimination for a time because of the lack of ground-truth data. Fortunately, with the increase of satellite resolution, it is possible to extract the features of detection results through the statistical analysis of the image features or mathematical transformation. In [35], the objects with a closed boundary are discriminated by its norm of gradients (NG), so an image window with a fixed size is chosen and its NG is extracted as the input of a cascaded support vector machine (SVM) classification. In [36], some pixels considered to be sea clutter are refused with the removal of the azimuth ambiguities and next the potential target pixels are gathered in clusters. The discrimination stage by means of kernel principal component analysis (PCA) removes sea clutter pixels from target pixels in [37], and this method proves to be superior to the classic CFAR algorithm by the experimental results of real data. Generally speaking, in the early stages of ocean monitoring, there were not enough measurements available to distinguish the targets and false alarms, partly due to the low resolution of SAR. The development of SAR provides the possibility of precise judgment, requiring features extracted from the image chips. Xu L presents a comprehensive comparison of classification techniques for oil-spill detection in SAR images [38]. The performance of the classifier using artificial neural network needs to be improved because of the classifier overfitting caused by too little data used to train the classifier. In our experiments, the classifier can make a good distinction between the true targets and the false alarms, which benefits from the training of a large number of SAR ship images. As the choice of features has an impact on the performance of discrimination, neural network becomes a superior tool because of its power in feature extract compared to some traditional methods with hand-crafted features. In [39], a framework named Sea-Land Segmentation-based Convolutional Neural Network (SLS-CNN) is proposed for ship detection. The SLS-CNN detector is combined with the use of saliency computation and distinguishes true ships from false alarms on the basis of Spectral Residual (SR) map. A modified Faster R-CNN based on CFAR algorithm for SAR ship detection is proposed in [40]. This method gains a good performance of detection at the expense of much more training time and requires a lot of data to train network, which may limit the use of this method. The document [41] presents a method categorizing ship targets from SAR images using texture features in artificial neural networks (TF-ANN). The TF-ANN method selects an appropriate texture feature for SAR images and uses the feature as the input of neural network to extract ship pixels from sea ones. Finally, in post-process step morphological filters are used to make the results much clearer. Therefore, in this paper we use a convolution neural network to separate false alarms from true targets, and the result shows that the use of neural networks in high-resolution images to remove false alarms is a reliable choice. Our work confirms the effectiveness of neural network for false alarms elimination in SAR images.

The Gaofen-3 satellite launched in 2016 by China aims at maritime surveillance, water environment monitoring, disaster prediction and so on, which is able to run in twelve different modes [42]. This paper researches the land masking strategy and some distributions to model sea clutter better with the purpose of ship detection in GF-3 SAR images. To boost the performance of the traditional CFAR-based detector, we use new methods in the stage of land masking and discrimination, compare quite a few distributions to make the prescreening stage much more efficient and accurate. The proposed detection method is mainly made of four parts: land masking with FCN, the sea clutter distribution analysis for GF-3 SAR images, the CFAR detection and the discrimination using neural network. The paper is organized as follows: details of the proposed method are listed in Section 2. Section 3 shows the experiments on several GF-3 SAR images to confirm the accurateness and effectiveness of this detection approach. Section 4 concludes the whole essay.

## 2. Methods

### 2.1. Overall Scheme Based on Sea Clutter Analysis and Neural Networks

**Figure 1:**
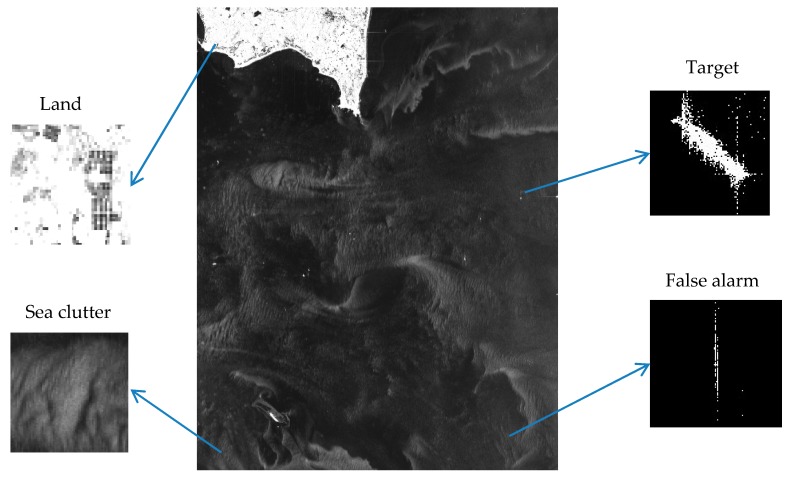
A GF-3 SAR image containing land, several ships and sea clutter.

**Figure 2:**
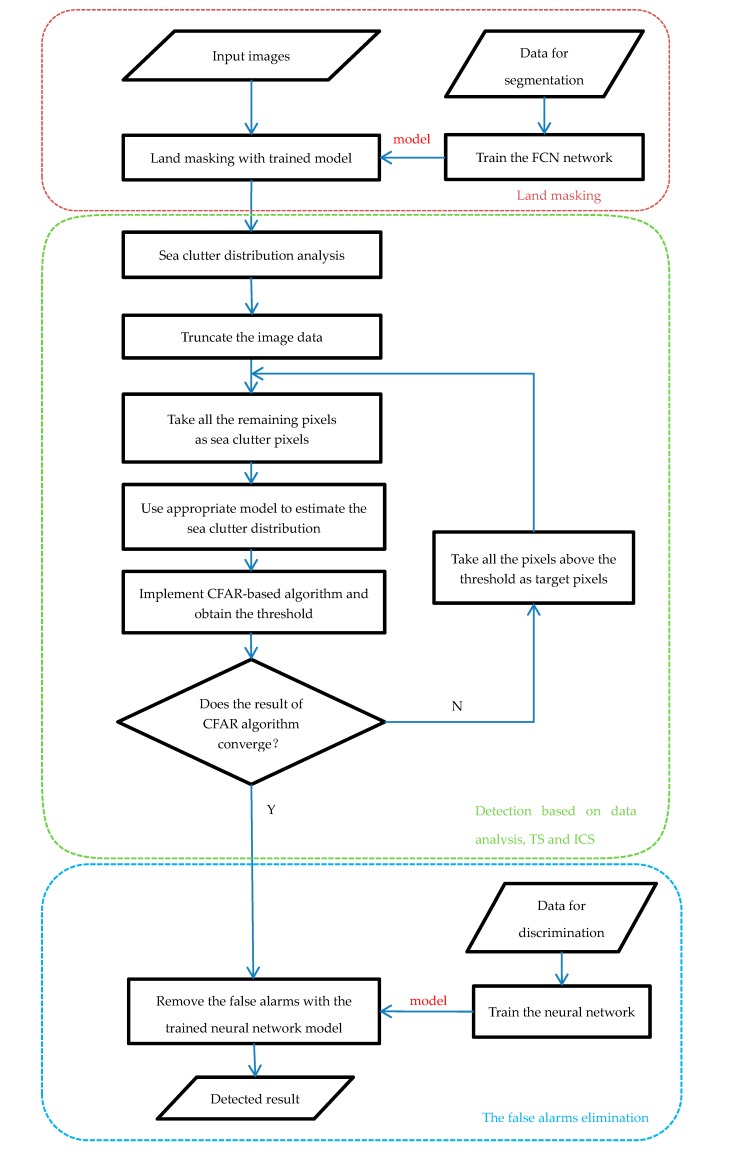
Workflow of our method for ship detection.

The core of ship detection in SAR images is modeling the sea clutter to find the bright target pixels. Figure 1 illustrates a GF-3 SAR image containing land, several ships and sea clutter. It clearly shows that the ships in the image are brighter than the sea clutter and so is the land, thus it is necessary to carry out land masking firstly, and implement the ship detection based on the amplitude values of pixels in the next step. Our proposed method for ship detection consists of four steps: the land masking with fully convolutional network, the analysis and selection of distribution model of sea clutter in GF-3 SAR images, the CFAR detection using truncated statistic and iterative censoring scheme, and the discrimination stage based on deep convolutional neural network to remove false alarms. Figure 2 illustrates the workflow of our method.

- Step 1, the land masking with fully convolutional network. As a method of deep learning, the fully convolutional network is employed for land masking to reduce false alarms in the course of detection.
- Step 2, the analysis and selection of distribution model for sea clutter. Through the analysis of the sea clutter distribution model, the probability distribution suitable for real applications is selected to compute an appropriate threshold which will improve the accuracy of the detection. In view of the use of iterative censoring-based methods, the efficiency should be an important factor to consider because the process of parameter estimation may be performed multiple times.
- Step 3, the CFAR detection using truncated statistic and iterative censoring scheme. In this step, truncated statistic is utilized to reduce the interference of potential target pixels when estimating the parameters and the iterative censoring scheme is a strategy to obtain the optimal threshold gradually so as to improve the performance of the result iteratively.
- Step 4, the discrimination stage to remove false alarms. Some bright non-target pixels are also likely to be detected as false alarms according to the basis of CFAR algorithm, thus a neural network is introduced to eliminate them. As the neural network is able to extract the appropriate features automatically, it is a powerful tool to increase the accuracy of discrimination.

### 2.2. Land Masking with Fully Convolutional Network

Land masking is an important phase in the process of ship detection. As is vividly revealed in Figure 1, land is much brighter than the sea clutter, which could interfere with the CFAR detector based on variant brightness of pixels.

**Figure 3:**
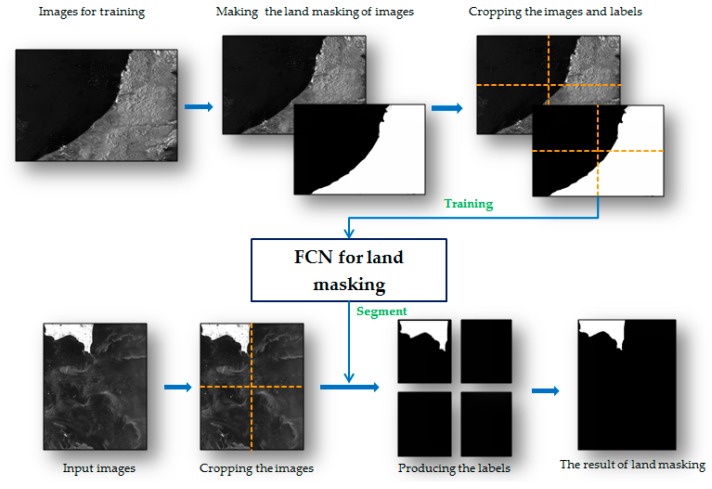
The process of land masking with FCN.

The fully convolutional network applies deep learning method in the field of semantic segmentation [15]. FCN is implemented with a deep learning framework named Caffe and popularizes the use of end to end convolutional networks for semantic segmentation. When using this method for image segmentation, training is needed to generate a competent model for new task. Figure 3 subtly depicts the process of using FCN for land masking. This method is divided into three steps.

Firstly, making training set. Some other GF-3 SAR images are required to adjust the model, in which step sea-land segmentation labels of images are made manually.

Second, the training of FCN network. The training set of SAR images and corresponding labels are cut into 500 × 500 image chips, which feed a successive fully convolutional network to generate a new model for sea-land segmentation.

Thirdly, land masking using trained model. The SAR images to be detected are divided into small chips which have the same size as training set. After that the trained network takes these image chips as input and produces their labels. Eventually, the cropped labels are put together to obtain the sea-land segmentation result.

We construct the FCN architecture for sea-land segmentation, and then train it with GF-3 SAR image blocks. The architecture of the network is shown as Table 1. It has ten convolution layers (C1–C10), five maximum pooling layers (P1–P5), and three deconvolution layers (D1–D3). It is necessary to point out that C8–C10 generate different scales of segmentation results, and D1–D3 restore them to the size of input images respectively.

**Figure 4:**
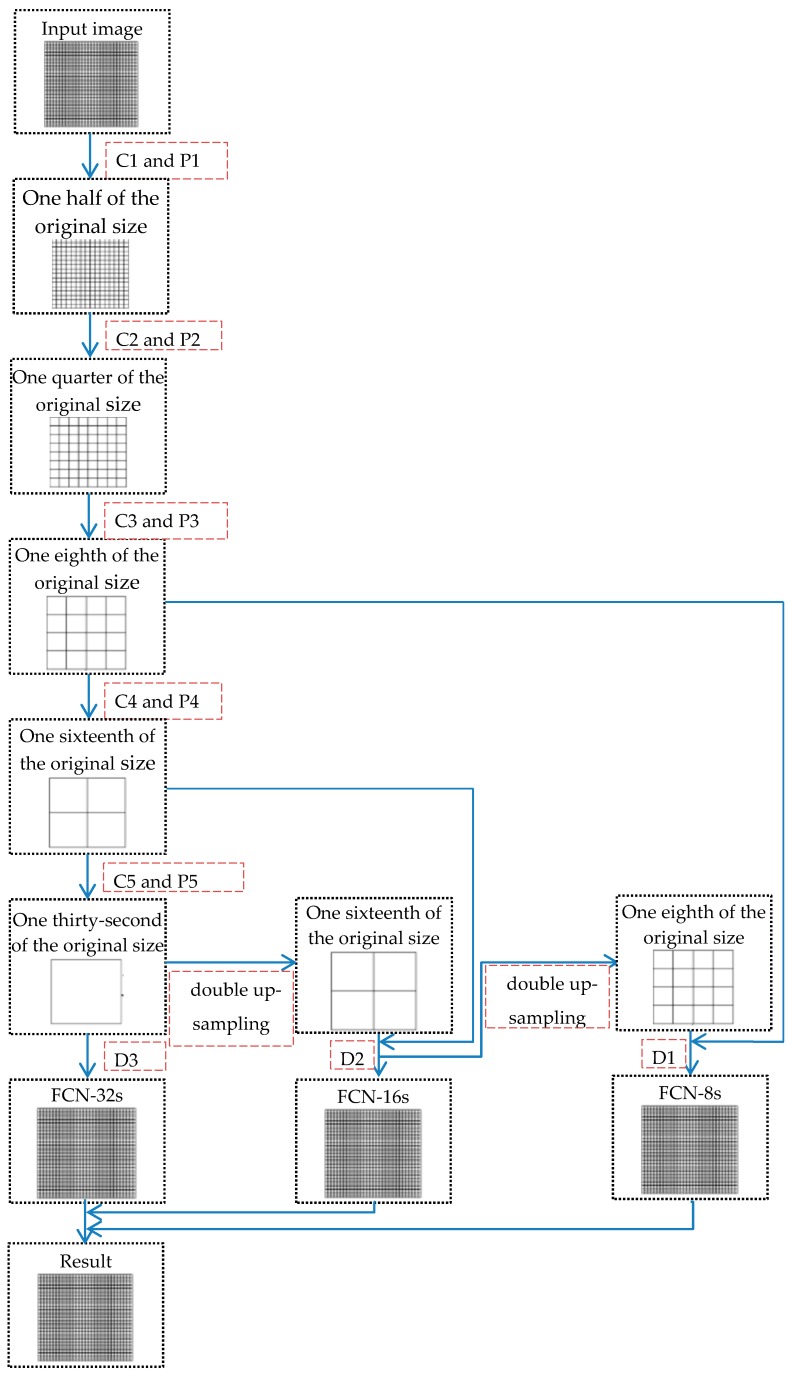
The training process of FCN.

The training is divided into three stages: first, get the segmentation results with C10’s output and train the network by comparing them with the input labels; second, get the segmentation results with C9’s output and fuse them with the results of the last stage to obtain the fusion results for training network; at last, generate the segmentation results with the output of C8 and combine them with the results of stage two to complete the training of the whole FCN network. The training process is shown as Figure 4.

The stage of land masking can significantly reduce the running time of the ship detection and boost the detector's precision by removing the interference of land pixels. After obtaining the sea-land labels, ship detection is only conducted in the scope of the pixels labeled as sea.

### 2.3. Analysis for the Distribution of Sea Clutter in GF-3 SAR Imagery

When CFAR-based algorithms are utilized to detect ships in the SAR images, it is worth noting which distribution should be chosen to model sea clutter. In the applications of high resolution SAR radar, the distribution of sea clutter is considered not to match the Gaussian form. In fact, it can be better described by Rayleigh distribution, Gamma distribution and the K-distribution [2,4,24,43]. Further, the performance of detection is bound up with the model of sea clutter, which determines the speed and accuracy of the detection.

It is widely accepted that the K-distribution is a good model for sea clutter in high resolution radar imagery. The probability density function (PDF) of K-distribution is defined as follows [44]:

(1) $$f_{X}(x)=\frac{2}{a\Gamma (v)}{\left(\frac{x}{2a}\right)}^{v}K_{v-1}(\frac{x}{a})\;\;x\geq 0,v>1,a>0$$

where x is the amplitude of sea clutter, v is the parameter determining the shape of the K-distribution, a is the scale parameter, Γ(⋅) is the gamma function, and $K_{v-1}(\cdot )$ is the modified Bessel function of order v−1. The cumulative distribution function (CDF) of x is defined as the integral of Equation (1). When a set of sea clutter pixels ${\left\{x_{i}\right\}}_{i=1}^{N}$ is given, a and v can be estimated with moment estimation according to Equations (2) and (3) [45,46,47]:

(2) $$\hat{v}={\left(\frac{{\hat{m}}_{4}}{2{\hat{m}}^{2}{}_{2}}-1\right)}^{-1}$$

(3) $$\hat{a}=\frac{1}{2}\sqrt{\frac{{\hat{m}}_{2}}{\hat{v}}}\;$$

where ${\hat{m}}_{2}$ and ${\hat{m}}_{4}$ are the 2-order and 4-order moment of sample pixels respectively. After the parameters in the equation are estimated, the corresponding threshold T can be calculated with a given false alarm rate $p_{f}$:

(4) $$p_{f}=1-\int_{0}^{T}f_{X}(x)dx$$

Similar to K-distribution, the gamma distribution is also a good model for sea clutter and has the advantage of being much easier to deal with. Suppose that a random variable x follows the gamma distribution, and its PDF can be computed as the following equation:

(5) $$f_{X}(x)=\frac{1}{x}{\left(\frac{vx}{\mu }\right)}^{v}\frac{1}{\Gamma (v)}\exp(-\frac{vx}{\mu })\;\;x\geq 0,v>0,\mu >0$$

where x is the amplitude of sea clutter pixels, μ is the mean value of x, v is the parameter determining the shape of the gamma distribution, and Γ(⋅) is the gamma function. The parameters can be estimated according to Equations (6) and (7):

(6) $$\hat{\mu }=\frac{1}{N}\sum_{i=1}^{N}x_{i}$$

(7) $$\hat{v}=\frac{{\hat{\mu }}^{2}}{\frac{1}{N-1}\sum_{i=1}^{N}{\left(x_{i}-\hat{\mu }\right)}^{2}}$$

where *N* is the number of all pixels. It is the same as the K-distribution mentioned before that the threshold *T* can be calculated based on a given *P_f_*.

The CFAR detector based on Rayleigh distribution is employed universally because of its concision and good performance when modeling homogeneous areas. The probability density function of Rayleigh is defined as:

(8) $$f_{X}(x)=\frac{x}{\sigma^{2}}\exp(-\frac{x^{2}}{2\sigma^{2}})\;\;x\geq 0,\sigma >0$$

where x is the amplitude of sea clutter pixels and σ is a parameter related to the 2-order moment of sample pixels, it can be estimated as:

(9) $${\hat{\sigma }}^{2}=\frac{1}{2N}\sum_{i=1}^{N}x_{i}{}^{2}$$

According to the equations listed, the threshold of pixel values is obtained and separates targets from sea clutter. It is worth noting that although the three distributions mentioned are all practical in CFAR-based algorithms, it is unlikely to be appropriate to choose one distribution arbitrarily. For one thing, there is no denying that the calculation process of the Rayleigh distribution is the simplest among the three distributions, while the gamma distribution is more complex than Rayleigh, and K-distribution requires the most complicate computational process at the expense of a long running time. For another, the three distributions are to be tested with the real GF-3 SAR images.

In order to compare the performance of the three distributions on modeling the GF-3 data, we use the images after land masking to estimate the parameters of these distributions. The accuracy of the fitting of sea clutter histogram and the calculated error will be considered as influencing factors determining the merits of the distributions. So as to ensure that the pixels used to evaluate the performance of distributions do not mix with target pixels, we divide the GF-3 SAR images into many patches and calculate the mean values of them. The patch with the lowest mean value is considered to merely consist of sea clutter, which is suited to estimate the parameters of distributions and evaluate their performance.

**Figure 5:**
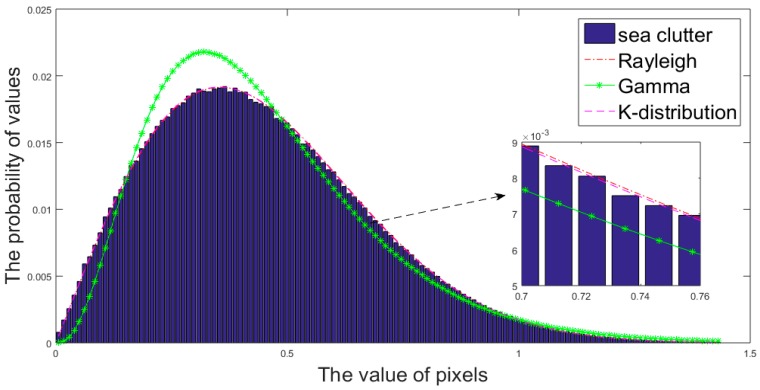
The histogram of sea clutter and three corresponding distribution curves.

Figure 5 indicates the performance of three distributions in estimating the probability density function of sea clutter based on a GF-3 SAR image with the resolution of 5 m. As the chart shows, the performance of both K-distribution and Rayleigh is certain to meet expectation intuitively. By calculating the cross entropy and the mean squared error (MSE) of the three distributions with the real SAR data, the result effectively clarifies the conclusion that Rayleigh has a property close to K-distribution and both of them are evidently better than the gamma distribution. Equations (10) and (11) show how to compute the cross entropy and MSE respectively:

(10) $$H(p,q)=\sum_{i=1}^{n}p_{i}\cdot \log(\frac{1}{q_{i}})$$

(11) $$MSE=\frac{1}{n}\sum_{i=1}^{n}{\left(q_{i}-p_{i}\right)}^{2}$$

In the above formulae, we divide the intensities of the pixels into 256 intervals and n is the number of intervals; the frequency distribution of sea clutter pixels is p, the distribution estimated from the read data is *q*, *p_i_* and *q_i_* represent the values of real data and estimated distribution in the *i*-th interval, respectively.

Table 2 shows the error and running time of the three distributions on modeling sea clutter, and the averaged running time is obtained by MATLAB programming on a computer with Inter Core at 3.2 GHz. With respect to the error, K-distribution achieves the best performance, which is in line with the relevant theory [2,21]. Nevertheless, it is worth noting that the image slices used to estimate properties of the three distributions are roughly 3000 × 3000 pixels in size, which could be much larger when processing a whole GF-3 SAR image. In addition, the efficiency is an important factor to consider because the parameter estimation may be performed multiple times when the iterative censoring-based methods are utilized. Therefore, the cost of time may impede the use of K-distribution in the case of rapid detection or limited computational resources. Under these circumstances, Rayleigh has the ability to substitute for K- distribution thanks for its efficiency and similar performance to K-distribution. As vividly revealed in the table, the errors of Rayleigh are almost the same as K-distribution's, but the running time of latter is twenty times more than the former’s.

From what has been discussed above, we can safely draw the conclusion that K-distribution has the advantage of precise description of the sea clutter at the expense of much running time, and Rayleigh distribution may be more practical for GF-3 SAR images because of its faster speed and no significant performance deterioration.

### 2.4. Iterative CFAR with Truncated Statistic

The CFAR algorithm sets an appropriate threshold to detect target pixels, which requires an accurate estimate of the distribution for the sea clutter. Therefore, there are two key points worthy of attention in the phase of prescreening. One issue is the choice of pixels which are used to estimate the parameters of distribution. The second point of detection is the approach to setting a suitable threshold according to the statistical distribution, which aims at seeking for unusually bright pixels.

With regard to the SAR images with land masking, the pixels that need to be detected consist of sea clutter and targets. In order to obtain an accurate model of sea clutter, the target pixels should be removed out to eliminate their interference. According to the proposed method in [25], when the region being detected contains some non-clutter sections, pixels truncation is a powerful tool to get rid of possible targets. This method demands that the pixels of image are sorted by the values, and the pixels with large values are considered to be potential targets. The truncation of the data requires an appropriate ratio so as not to remove lots of sea clutter pixels with large values. In general, the detection of heterogeneous sea clutter demands for cropping the image, and each image chip should not exceed the size of 3200 pixels square [44]. Taking into account that most of the detected ships are no more than three hundred meters, thus each ship's pixels are generally less than several thousand when the resolution of SAR is 3 m. This number should be smaller when the resolution is 8 m.

Therefore, compared to millions of pixels to be detected in every SAR image chip, the number of possible ship pixels can barely take up a small portion, in the case that the ships are not travelling densely. Consequently, in this essay we set the ratio of truncated statistic to 0.995 to exclude the potential impact of possible target pixels on the basis that the majority of sea clutter pixels are reserved, which cannot be smaller to avoid damaging the statistic characteristics of sea clutter.

Once a false alarm rate is given, the threshold can be computed based on the distribution model selected before. Although there are some theories about false alarm rate settings, this is still an important issue that a higher threshold often results in missed detection of targets and lower thresholds regard more sea clutter as targets. Since determining the threshold of detection directly is reckless to some extent, it is wise to update the threshold of detection iteratively. The research in [27] illustrates that the iterative censoring scheme is an outstanding tool to determine the appropriate detection threshold and improve the detection accuracy by detecting the target pixels gradually.

The ICS strategy generally consists of three steps: first, take all the remaining pixels as sea clutter to estimate the parameters of the distribution; second, CFAR detector is utilized to obtain the potential target pixels; third, examine whether the threshold is convergent, if not, remove out the potential target pixels and repeat the previous steps. At last, this method will provide a threshold as the boundary between the background and the targets.

**Figure 6:**
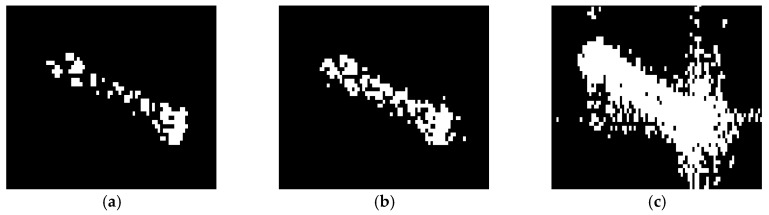
Detected results of a true target. (**a**) Detected result without TS; (**b**) Detected result without ICS; (**c**) Detected result with TS and ICS.

The iterative censoring scheme converges quickly and the results of the detection are continuously optimized as the iterations are carried out. Figure 6 vividly verifies that iterative CFAR with truncated statistic is able to make the ships easy to identify, which provides convenience for extracting the features of connected domains and eliminating the false alarms.

### 2.5. False Alarms Discrimination with a Neural Network

Discrimination is an indispensible stage, aiming at reducing the number of false alarms. The CFAR algorithms detect the unusually bright pixels, but some azimuth ambiguity and bright lines are also detected out. The discrimination stage extracts the features of true targets and separates them from a variety of false alarms. In this phase, the isolated bright pixels are firstly removed out, then all the potential targets are classified by a neural network and the discrimination results will be marked on the origin SAR images.

**Figure 7:**
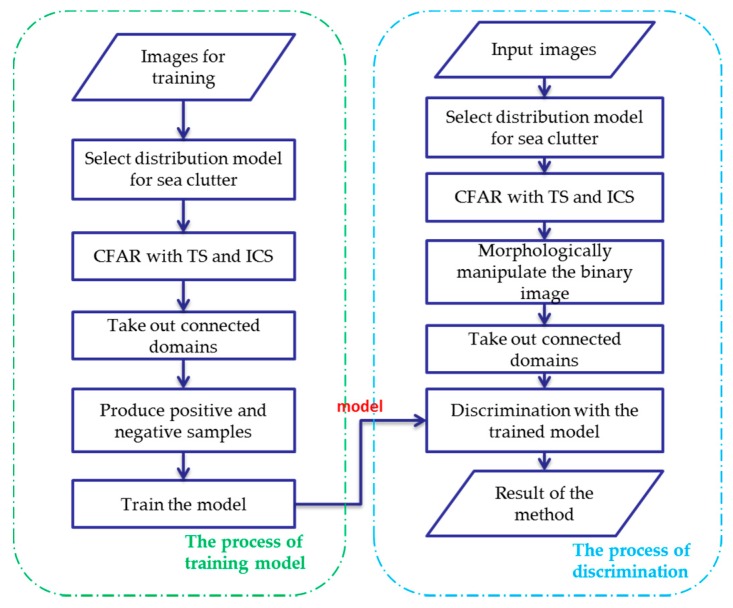
The discrimination stage with deep convolutional neural network.

The first step is to morphologically manipulate the binary image which has been detected by CFAR algorithm such as corrosion and expansion, so as to remove those isolated false alarms whose size is too small. Next, the connected domains in the binary image are taken out one by one, and then attach each of them to the center of the image chips at the same size of 500 pixels square. After that, use some other SAR images to produce training set with the same size as image chips in the previous step and train a neural network. In order to improve the recognition ability of the neural network, the samples used to train the model should include different kinds of ship targets and various forms of false alarms as positive samples and negative samples, respectively. In addition, the positive and negative samples should be rotated to obtain images at different angles to eliminate the interference of various orientations. Finally, the trained network is applied to judge whether the input image chips are targets or false alarms. According to the results given by the trained network, the connected domains containing ship are marked on the original image. We construct a neural network for false alarms elimination, and the architecture of it is shown as Table 3. There are two convolution layers (C1, C3), two maximum pooling layers (P2, P4), and three fully-connected layers (F5, F6, F7). This network is trained by SAR image blocks with their labels, which are ships or not. Figure 7 shows the whole process of discrimination.

## 3. Experiments and Discussion

### 3.1. Experimental Data

Three GF-3 SAR images to be detected are employed in the experiment, and the specific information about the image is listed in Table 4. The UFS is short for ultrafine strip-map. It is one of the running modes of the GF-3, under which mode the signal has two channels with the horizontal and the vertical polarization. As shown in the table, these images are all single-looks without multi-looks processing. Rng and Az are short for range and the azimuth directions. TIFF as the short of tag image file format is a flexible kind of bitmap format of images, which is widely used in SAR imagery.

**Figure 8:**
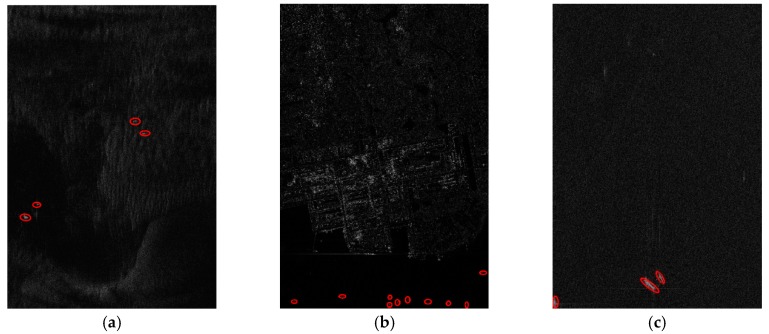
The sub-images to be detected with ground truth marked. (**a**) The sub-images #1 from image #1; (**b**) The sub-images #2 from image #2; (**c**) The sub-images #3 from image #3.

To show the detected results better, we crop the three images and extract three sub-images. These sub-images are cropped from image #1, #2 and #3 in several, with the same size of 4467 × 6446 (Rng × Az). Some of the real objects identified by professional image interpreters are marked on the images with red thick circles in Figure 8.

### 3.2. Sea-Land Segmentation Results of the Proposed Method

**Figure 9:**
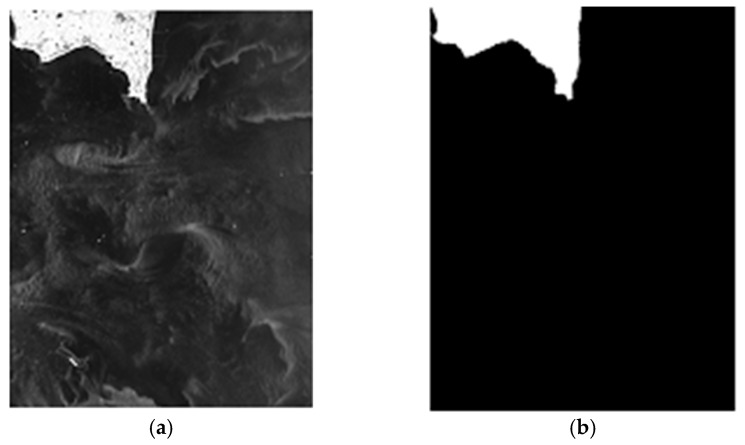

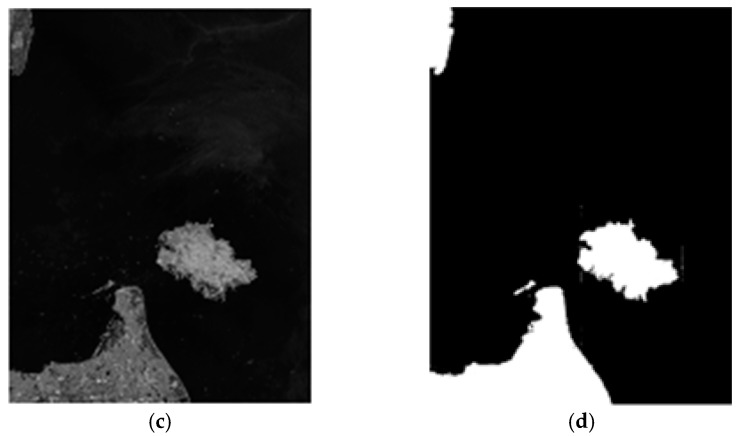
Sea-land segmentation results of the proposed method. (**a**,**b**) are Image #1 and the land masking result corresponding to it; (**c**,**d**) are Image #2 and the land masking result corresponding to it.

We implement the proposed method of sea-land segmentation on a computer equipped with a NVIDIA K40 board. The two images (image #1 and #2) to be segmented and the segmentation results of the proposed method are shown in Figure 9. The highlighted region is segmented to be the land and the rest is the ocean. As revealed in the figure, the sea-land segmentation results are fundamentally consistent with the actual geographical situation. Compared with the segmented results by human supervision, the mismarks merely account for 0.141‰ of the total pixels in Image #1 and 0.453‰ of the total pixels in Image #2. In contrast with the sea-land segmentation results by the maximum entropy (ME) and OTSU algorithms [11,12], the land masking method using FCN has an evident advantage in regard to accuracy and some other indices, which illustrate the superiority of this method for GF-3 SAR images (see Table 5). In this experiment, ‘false positive’ refers to the sea clutter pixels judged as land pixels and ‘false negative’ refers to the opposite meaning. The pixels marked as land will not participate in sea clutter analysis or CFAR-based detection, ensuring the accuracy of distribution modeling and reducing the number of false alarms. Although the correct rates of ME and OTSU are both more than 90 percent, it is worth noting that their false negative rates are much higher than our method, which means that there will be a large number of land pixels used for ship detection. The inadequacy of ME and OTSU may overwhelm the significance of land masking.

### 3.3. Detected Results of the Method

The detection is implemented with MATLAB programming on a computer equipped with an Intel Core CPU operating at 3.2 GHz. The detected results are shown as binary indication images, and the pixels with the value of one represent the potential targets, while pixels with the value of zero are detected as sea clutter.

This process uses a Rayleigh distribution to estimate distribution parameters of the sea clutter, and the detected results reveal that the Rayleigh distribution can highlight the potential target well at a cost of little time when processing the GF-3 SAR images. In particular, the Rayleigh distribution shows a greater advantage due to the use of iterative censoring-based methods, which significantly reduces the running time.

**Figure 10:**
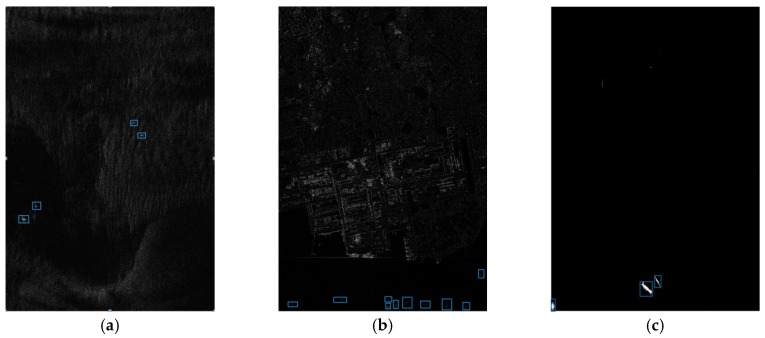
Results of the whole method on sub-images #1 to #3. (**a**) Result of sub-image #1; (**b**) Result of sub-image #2; (**c**) Result of sub-image #3.

We implement the discrimination stage on a computer with a NVIDIA K40 board, and the results are marked on the origin images as Figure 10:

It can be concluded from Table 6 that the running time of the proposed method is able to meet the accuracy requirement at a cost of little running time, which benefits from the sea clutter distribution analysis for GF-3 SAR images and the simple distribution model adopted in the process of CFAR detection. Compared with the prescreening, the land masking with FCN and the discrimination using neural network consume most of the running time, which may be a weakness of deep convolutional networks. However, the difference between various ship detectors usually reflects in the capability to exclude the impact of land and eliminate the false alarms. The use of neural networks significantly improves the performance of land masking and automatically extracts the characteristics of the ships so as to effectively reject false alarms. Therefore, it is worth spending some time on the preprocessing and discrimination to make the detector much more robust.

### 3.4. Comparison with Recently Developed Works Using Neural Network

In this section, we compare our method with two state-of-the-art SAR ship detection methods using neural networks, including SLS-CNN described in [39] and TF-ANN in [41]. Both methods focus on feature extraction and feed neural networks with image blocks containing potential ships. Their detected results are based on heat map of feature computation, so we show the heat map and plot the detected boxes in it to show the positions of the detected ships obtained by SLS-CNN and TF-ANN.

**Figure 11:**
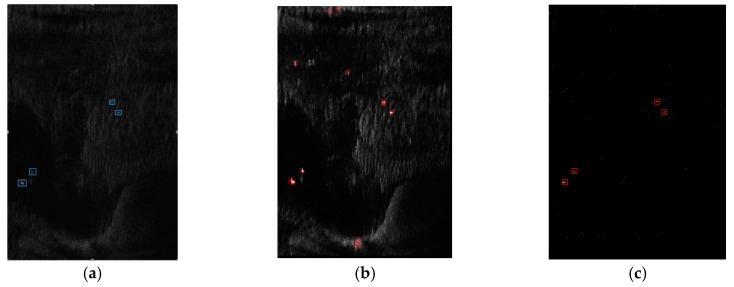
Results of the various methods on sub-images #1. (**a**) Detected result of the proposed method; (**b**) Detected result of the SLS-CNN method; (**c**) Detected result of the TF-ANN method.

Figure 11 compares the detected results of SLS-CNN, TF-ANN and our method on sub-image #1. It can be seen that SLS-CNN can detect the true ships, however, this method generates some false alarms caused by noise, and these false alarms can’t be discriminated by the neural network because of their similar shape to the true ships. On the other hand, a single ship may be detected multiple times as the result of the sliding search window, which is not taken into consideration by this method. The TF-ANN performs well in removing false alarms thanks to the multiple features extracted from the input SAR images and the artificial neural network fed with the most appropriate feature, nevertheless, it takes a lot of time to calculate a variety of features (see Table 7), which may impede the use in real-life ship detection.

### 3.5. The Performance of the Generalization of the Method

In order to analyze the performance of the generalization of the proposed method, we apply this method in some TerraSAR-X images with spatial resolution of 1 m. Here, the TerraSAR-X images include Singapore in April 2010.

**Figure 12:**
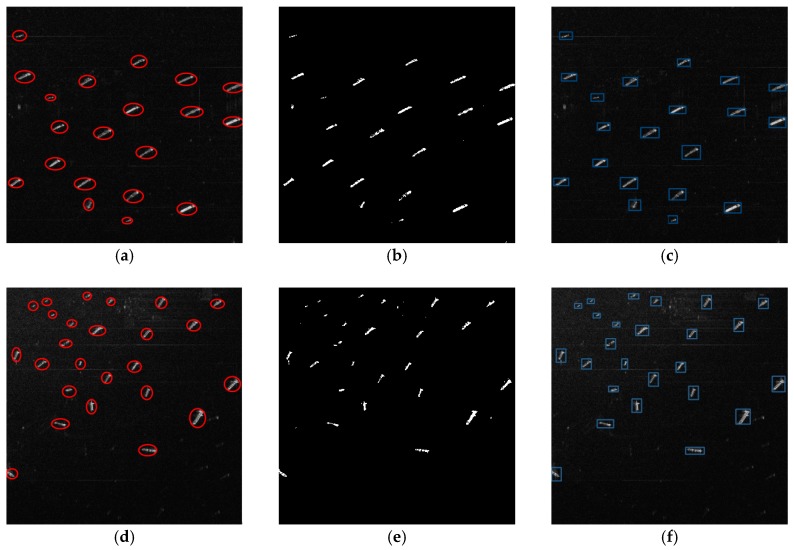
Results of the method on TerraSAR-X images. (**a**) The sub-image #4 from TerraSAR-X image; (**b**) The binary indication image of sub-image #4; (**c**) Result of sub-image #4; (**d**) The sub-image #5 from TerraSAR-X image; (**e**) The binary indication image of sub-image #5; (**f**) Result of sub-image #5.

To show the detected results better, we crop the TerraSAR-X images and take out two sub-images from them. These sub-images have the same size of 2048 × 2048 (Rng × Az). Some of the real objects identified by professional image interpreters are marked on the images with red thick circles, and the detected results are marked by blue rectangles. The origin sub-images, the binary indication images and the detected results of our method are shown in Figure 12.

It can be seen from the above figures that all the ship targets have been detected, and the deep learning network can eliminate the false targets from the actual ships at the stage of discrimination. This experiment shows that our method also has a good performance on TerraSAR-X images and proves the generalization ability of this method.

## 4. Conclusions

This paper proposes a method for ship detection in GF-3 SAR images based on sea clutter distribution analysis and a deep convolutional neural network. The method consists of four parts: land masking with FCN, sea clutter distribution analysis in GF-3 SAR images, the iterative CFAR-based prescreening with TS and the discrimination with a neural network. Our experiments show that the use of the fully convolutional network gives encouraging performance at the stage of sea-land segmentation, which lays a good foundation for the subsequent steps. In the prescreening phase, a Rayleigh distribution may be a better choice to model the sea clutter when the speed and accuracy are both taken into account. The use of truncated statistics, which separates background pixels from the true targets pixels, also contributes to the improvement of sea clutter description. On the basis of sea clutter modeling, we apply an ICS method to update the parameters of detection iteratively. Finally, a neural network is used for discrimination to remove false alarms, which shows prior ability to extract image features. Since the Rayleigh distribution is used as an alternative to the K-distribution in the course of the detection process, this method is able to quickly generate the detected results of GF-3 SAR images, while the FCN for land masking and neural network for discrimination greatly enhance the reliability of the entire detection system. The detected results illustrate the effectiveness of the proposed detection strategy, which provides an efficient and effective method for ship detection in GF-3 SAR images.

## Figures and Tables

**Table 1:** The architecture of FCN for sea-land segmentation.

Layer	Size of Filter	Stride	Feature Dimensions	Layer	Size of Filter	Stride	Feature Dimensions
Input			1 × 500 × 500				
C1	3 × 3	1	64 × 698 × 698	P4	2 × 2	2	512 × 44 × 44
P1	2 × 2	2	64 × 349 × 349	C9	1 × 1	1	2 × 44 × 44
C2	3 × 3	1	128 × 349 × 349	D2	32 × 32	16	2 × 512 × 512
P2	2 × 2	2	128 × 175 × 175	C5	3 × 3	1	512 × 44 × 44
C3	3 × 3	1	256 × 175 × 175	P5	2 × 2	2	512 × 22 × 22
P3	2 × 2	2	256 × 88 × 88	C6	7 × 7	1	4096 × 16 × 16
C8	1 × 1	1	2 × 88 × 88	C7	1 × 1	1	4096 × 16 × 16
D1	16 × 16	8	2 × 512 × 512	C10	1 × 1	1	2 × 16 × 16
C4	3 × 3	1	512 × 88 × 88	D3	64 × 64	32	2 × 512 × 512

**Table 2:** The performance of three distributions on modeling sea clutter in GF-3 SAR imagery.

Index	Gamma	Rayleigh	K-Distribution
Cross entropy for image-slice #1	4.6899	4.6681	4.6661
Cross entropy for image-slice #2	5.0953	5.0710	5.0699
Cross entropy for image-slice #3	5.1325	5.1060	5.1053
MSE for image-slice #1	2.6117	2.2287	2.1970
MSE for image-slice #2	2.4358	2.0260	2.0028
MSE for image-slice #3	2.3157	1.8777	1.8649
Averaged running time per slice (ms)	162	43	871

**Table 3:** The architecture of neural network for discrimination.

Layer	Feature Maps	Size of Filter or Pool	Feature Dimensions
Input		128 × 128	16,384
C1	6 × 124 × 124	5 × 5	92,256
P2	6 × 62 × 62	2 × 2	23,064
C3	16 × 58 × 58	5 × 5	53,824
P4	16 × 29 × 29	2 × 2	13,456
F5	1024		1024
F6	32		32
F7	2		2
Output			2

**Table 4:** The information about the three GF-3 SAR images employed in the experiment.

Parameter	Image #1	Image #2	Image #3
Region	South China Sea	South China Sea	South Atlantic Ocean
Nominal Resolution(m)	3	3	3
Image mode	UFS	UFS	UFS
Number of looks (Rng × Az) (m)	1 × 1	1 × 1	1 × 1
Center Frequency (GHz)	5.4000	5.4000	5.4000
Center look angle (degree)	37.1600	37.1600	41.8200
Look direction	Right	Right	Right
Size (Rng × Az) (pixel)	18,230 × 22,439	18,233 × 32,757	20,850 × 22,566
Pixel width (Rng × Az) (m)	1.1242 × 1.6135	1.1242 × 1.6134	1.1242 × 1.6797
Product level	1	1	1
Product format	TIFF	TIFF	TIFF

**Table 5:** Comparison of land masking results of various methods on Image #1.

Parameter	Our Method	ME	OTSU
Correct rate	0.9999	0.9730	0.9392
False positive rate	0.0000	0.0152	0.0040
False negative rate	0.0001	0.0118	0.0568

**Table 6:** The running time of each stage and detected results of the proposed method.

Parameter	Sub-Image #1	Sub-Image #2	Sub-Image #3
Number of true targets	4	10	3
Running time of land masking	1.145 s	1.139 s	1.132
Running time of prescreening	0.83 s	0.91 s	0.90 s
Running time of discrimination	2.10 s	5.32 s	1.85 s
False alarms before discrimination	3	5	2
False alarms after discrimination	0	0	0
Number of detected true targets	4	10	3

**Table 7:** The cost time of various methods on the experiment data.

Method	Sub-Image #1	Sub-Image #2	Sub-Image #3
SLS-CNN	11.15 s	10.19 s	11.29 s
TF-ANN	564.05 s	572.51 s	569.66 s
Our method	2.93 s	6.23 s	2.75 s

## References

[1] Crisp D.J. (2004). The State-of-the-Art in Ship Detection in Synthetic Aperture Radar Imagery.

[2] Wackerman C.C., Friedman K.S., Pichel W., Clemente-Colón P., Li X. (2001). Automatic detection of ships in RADARSAT-1 SAR imagery. Can. J. Remote Sens..

[3] Rye A.J., Sawyer F.G., Sothinathan R. A workstation for the fast detection of ships. Proceedings of the 10th IEEE International Geoscience and Remote Sensing Symposium (IGARSS 1990).

[4] Ferrara M.N., Torre A. Automatic moving targets detection using a rule-based system: Comparison between different study cases. Proceedings of the 18th IEEE International Geoscience and Remote Sensing Symposium, 1998 (IGARSS 1998).

[5] Descombes X., Moctezuma M., Maître H., Rudant J.P. (1996). Coastline detection by a Markovian segmentation on SAR images. Signal Process.

[6] Peis I., Illán I.A., Martínez-Murcia F.J., Segovia F., Górriz J.M., Ramírez J., Lang E.W., Salas-Gonzalez D. MRI brain segmentation using hidden Markov random fields with alpha-stable distributions. Proceedings of the 2016 IEEE Nuclear Science Symposium, Medical Imaging Conference and Room-Temperature Semiconductor Detector Workshop (NSS/MIC/RTSD 2016).

[7] Ji K., Leng X., Fan Q., Zhou S., Zou H. An land masking algorithm for ship detection in SAR images. Proceedings of the 36th IEEE International Geoscience and Remote Sensing Symposium (IGARSS 2016).

[8] Biamino W., Borasi M., Cavagnero M., Croce A., Matteo L.D., Fontebasso F., Tataranni F., Trivero P. A ‘dynamic’ land masking algorithm for synthetic aperture radar images. Proceedings of the 35th IEEE International Geoscience and Remote Sensing Symposium (IGARSS 2015).

[9] Gu D., Xu X. A novel procedure for land masking in ocean-land segmentation from SAR images. Proceedings of the 2014 IEEE International Conference on Signal Processing, Communications and Computing (ICSPCC 2014).

[10] De Nicolás J.M., Moya D.M., Amores P.J., del Rey Maestre N., Humanes J.L.B. Segmentation techniques for land mask estimation in SAR imagery. Proceedings of the 2013 Fifth International Conference on Computational Intelligence, Communication Systems and Networks (CICSyN 2013).

[11] Niharika E., Adeeba H., Krishna A.S.R., Yugander P. K-means based noisy SAR image segmentation using median filtering and otsu method. Proceedings of the 2017 International Conference on IoT and Application (ICIOT 2017).

[12] Li A., Li Y., Wang T., Niu W. Medical image segmentation based on maximum entropy multi-threshold segmentation optimized by improved cuckoo search algorithm. Proceedings of the 2015 8th International Congress on Image and Signal Processing (CISP 2015).

[13] Xu L., Li J., Wong A., Wang C. (2014). A KPCA texture feature model for efficient segmentation of RADARSAT-2 SAR sea ice imagery. Int. J. Remote Sens..

[14] Symeonakis E. Modelling land cover change in a Mediterranean environment using Random Forests and a multi-layer neural network model. Proceedings of the 36th IEEE International Geoscience and Remote Sensing Symposium (IGARSS 2016).

[15] Long J., Shelhamer E., Darrell T. Fully convolutional networks for semantic segmentation. Proceedings of the Conference on Computer Vision and Pattern Recognition (CVPR 2015).

[16] Liu Z., Liu B., Guo W., Zhang Z., Zhang B., Zhou Y., Ma G., Yu W. (2017). Ship Detection in GF-3 NSC Mode SAR Images. J. Radas..

[17] Gao G., Kuang G., Zhang Q., Li D. (2007). Fast detecting and locating groups of targets in high-resolution SAR images. Pattern Recognit..

[18] Xin Z., Liao G., Yang Z., Zhang Y., Dang H. (2017). Analysis of distribution using graphical goodness of fit for airborne SAR sea-clutter data. IEEE Trans. Geosci. Remote Sens..

[19] Gao G., Shi G. (2017). CFAR ship detection in nonhomogeneous sea clutter using polarimetric SAR data based on the notch filter. IEEE Trans. Geosci. Remote Sens..

[20] Gigli G., Lampropoulos G.A. A new maximum likelihood generalized gamma CFAR detector. Proceedings of the 22th IEEE International Geoscience and Remote Sensing Symposium (IGARSS 2002).

[21] Izzo A., Liguori M., Clemente C., Galdi C., Bisceglie M.D., Soraghan J.J. (2017). Multimodel CFAR detection in foliage penetrating SAR images. IEEE Trans. Aerosp. Electron. Syst..

[22] Di Vito A., Galati G., Mura R. (1994). Analysis and comparison of two order statistics CFAR systems. IEE Proc. Radar Sonar Navig..

[23] Yu Y., Huang S.-J., Torre A. Development of an automatic target detection and characterisation system in polarimetric SAR images. Proceedings of the 15th IEEE International Geoscience and Remote Sensing Symposium (IGARSS 1995).

[24] Gagnon L., Oppenheim H., Valin P. (1998). R&D activities in airborne SAR image processing/analysis at Lockheed Martin Canada. Proc. SPIE Int. Soc. Opt. Eng..

[25] Tao D., Doulgeris A.P., Brekke C. (2016). A segmentation-based CFAR detection algorithm using truncated statistics. IEEE Trans. Geosci. Remote Sens..

[26] Tao D., Anfinsen S.N., Brekke C. (2016). Robust CFAR Detector Based on Truncated Statistics in Multiple-Target Situations. IEEE Trans. Geosci. Remote Sens..

[27] An W., Xie C., Yuan X. (2014). An improved iterative censoring scheme for CFAR ship detection with SAR imagery. IEEE Trans. Geosci. Remote Sens..

[28] Cui Y., Zhou G., Yang J., Yamaguchi Y. (2011). On the iterative censoring for target detection in SAR images. IEEE Geosci. Remote Sens. Lett..

[29] Gao G., Liu L., Zhao L., Shi G., Kuang G. (2009). An adaptive and fast CFAR algorithm based on automatic censoring for target detection in high-resolution SAR images. IEEE Trans. Geosci. Remote Sens..

[30] Pan Z., Liu L., Qiu X., Lei B. (2017). Fast Vessel Detection in Gaofen-3 SAR Images with Ultrafine Strip-Map Mode. Sensors.

[31] Kourti N., Shepherd I., Schwartz G., Pavlakis P. (2001). Integrating spaceborne SAR imagery into operational systems for fisheries monitoring. Can. J. Remote Sens..

[32] Vachon P.W., Adlakha P., Edel H., Henschel M., Ramsay B., Flett D., Rey M., Staples G., Thomas S. (2000). Canadian progress toward marine and coastal applications of synthetic aperture radar. Johns Hopkins APL Tech. Dig..

[33] Rey M.T., Campbell J., Petrovic D. (1998). A Comparison of Ocean Clutter Distribution Estimators for CFAR-Based Ship Detection in RADARSAT Imagery.

[34] Eldhuset K. (1996). An automatic ship and ship wake detection system for spaceborne SAR images in coastal regions. IEEE Trans. Geosci. Remote Sens..

[35] Dai H., Du L., Wang Y., Wang Z. (2016). A Modified CFAR algorithm based on object proposals for ship target detection in SAR images. IEEE Geosci. Remote Sens. Lett..

[36] Iervolino P., Guida R., Whittaker P. A novel ship-detection technique for Sentinel-1 SAR data. Proceedings of the 2015 IEEE 5th Asia-Pacific Conference on Synthetic Aperture Radar (APSAR 2015).

[37] Meng W., Ju T., Yu H. CFAR and KPCA for SAR image target detection. Proceedings of the 2010 3rd International Congress on Image and Signal Processing.

[38] Xu L., Li J., Brenning A. (2014). A comparative study of different classification techniques for marine oil spill identification using RADARSAT-1 imagery. Remote Sens. Environ..

[39] Liu Y., Zhang M., Xu P., Guo Z. SAR ship detection using sea-land segmentation-based convolutional neural network. Proceedings of the 2017 International Workshop on Remote Sensing with Intelligent Processing (RSIP).

[40] Kang M., Leng X., Lin Z., Ji K. A modified faster R-CNN based on CFAR algorithm for SAR ship detection. Proceedings of the 2017 International Workshop on Remote Sensing with Intelligent Processing (RSIP).

[41] Khesali E., Enayati H., Modiri M., Mohseni Aref M. Automatic Ship Detection in Single-Pol Sar Images Using Texture Features in Artificial Neural Networks. Proceedings of the International Archives of the Photogrammetry, Remote Sensing and Spatial Information Sciences 2015 (ISPRS 2015).

[42] Liu J., Qiu X., Hong W. Automated ortho-rectified SAR image of GF-3 satellite using Reverse-Range-Doppler method. Proceedings of the 36th IEEE International Geoscience and Remote Sensing Symposium (IGARSS 2016).

[43] Nouar N., Farrouki A. CFAR detection of spatially distributed targets in k-distributed clutter with unknown parameters. Proceedings of the 2014 22nd European Signal Processing Conference (EUSIPCO 2014).

[44] Henschel M.D., Rey M.T., Campbell J.W.M., Petrovic D. (1998). Comparison of probability statistics for automated ship detection in SAR imagery. Proceedings of SPIE.

[45] Farina A., Gini F., Greco M.V., Verrazzani L. (1997). High resolution sea clutter data: Statistical analysis of recorded live data. IEE Proc. Radar Sonar Navig..

[46] Blacknell D., Tough R.J.A. (2001). Parameter estimation for the K-distribution based on [z log(z)]. IEE Proc. Radar Sonar Navig..

[47] Iskander D.R., Zoubir A.M. (1999). Estimation of the parameters of the K-distribution using higher order and fractional moments [radar clutter]. IEEE Trans. Aerosp. Electron. Syst..

